# Modification of Susceptible and Toxic Herbs on Grassland Disease

**DOI:** 10.1038/srep30635

**Published:** 2016-09-16

**Authors:** Xiang Yao, Yubing Fan, Qing Chai, Richard D. Johnson, Zhibiao Nan, Chunjie Li

**Affiliations:** 1 The State Key Laboratory of Grassland Agro-ecosystems; College of Pastoral Agriculture Science and Technology, Lanzhou University, Lanzhou, Gansu 730020, China; 2 Department of Agricultural and Applied Economics, University of Missouri, Columbia, Missouri 65211, USA; 3 AgResearch Ltd., Grasslands Research Centre, Palmerston North, New Zealand

## Abstract

Recent research shows that continuous overgrazing not only causes grassland biodiversity to decline, but also causes light fungal disease. *Achnatherum inebrians* is susceptible to fungal diseases and increases in prevalence during over grazing due its toxicity to livestock. This study aimed to examine the effects of *A. inebrians* on biological control organisms and levels of plant diseases in overgrazed grasslands in northwestern China. The results showed that *A. inebrians* plants were seriously infected by fungal diseases and that this led to a high incidence of the mycoparasitic species *Ampelomyces quisqualis* and *Sphaerellopsis filum*. In addition, the fungivore, Aleocharinae, was found only in the soil growing *A. inebrians* rather than in the overgrazed area without *A. inebrians*. Overall, in an overgrazed grassland fenced for one year, disease levels in blocks without *A. inebrians* were significantly higher than those in blocks with *A. inebrians*. Our findings indicated that the disease susceptible, toxic *A. inebrians* can help control plant disease levels in overgrazed grasslands.

In recent decades, there has been serious overgrazing problems in grasslands of northwestern China, including the Qinghai-Tibetan Plateau and Inner Mongolia^1^,^2^,^3^. Studies have shown that overgrazing can cause a decline in the biodiversity of flora and fauna systems^4^,^5^ and reduce disease severity in grasslands^6^,^7^,^8^. Recently, fencing to reduce overgrazing has been applied to assist in restoring grasslands^9^,^10^. Subsequently, a question worth exploring is whether the disease on plants of an overgrazed grassland would still stay at a low level as the grassland is fenced.

In contrast to grassland degeneration, *Achnatherum inebrians* (drunken horse grass) grows well in overgrazed grasslands due to its toxicity to sheep, goats, cattle^11^ and horses^12^. It has been confirmed that the toxicity affecting some large grazing animals is from a symbiotic fungal endophyte (*Epichloë* species) in *A. inebrians*^13^. The drunken horse grass is perennial and can be found in typical grasslands of northwestern China, including Gansu province, Qinghai province, Tibetan autonomous region, Xinjiang Uygur autonomous region and some areas of Inner Mongolia. Some surveys have indicated that the area growing this grass was expanding, for example, in Xinjiang, *A. inebrians* occupied 400 000 ha in 1987 and had expanded to 533 000 ha by 1992^11^. This ongoing increase was mainly due to the overgrazing of the grasslands^11^, and the fact that *A. inebrians* can easily spread by seeds establishing bare land induced by burrowing activity of rodents in overgrazed grasslands^14^. Given the toxicity of the grass to livestock and its broad distribution, many studies have focused on the control and eradication of *A. inebrians*^15^,^16^,^17^. Recently, it was reported that *A. inebrians* could protect grassland biodiversity by deterring livestock grazing just like the function of wire fence^14^. However, to the best of our knowledge, the ecological impacts of this toxic, susceptible grass on grassland diseases are not well understood.

In contrast to low disease incidence in overgrazed grasslands, *A. inebrians* has been reported to be seriously infected by several fungal diseases present on leaves and flowering stems, specifically, rust (*Puccinia stipae-sibiricae*), powdery mildew (*Blumeria graminis*), stem smut(*Ustilago hypodytes*) and ergot (*Claviceps purpurea*), which resulted in the formation of numerous hyphe and spores^18^. In order to fully understand the ecological role of *A. inebrians* to assist recovery of overgrazed grassland, we investigated the following two questions: 1) can the hyphe and spores generated by fungal diseases present on leaves of *A. inebrians* support the abundance of mycoparasites and fungivorous arthropods? 2) Can the toxic, diseased *A. inebrians* influence the level of disease incidence when the grassland is fenced for restoration purposes?

## Results

### Disease indexes of plants in grasslands fenced or overgrazed for 18 years (fenced vs. overgrazed grasslands)

The presence of different plant species was recorded. Forage plants are listed first, followed by other herbs (Table 1). The percentage of dry weight for the different species was also calculated (Supporting Information S1). In total, 14 plant species were listed in Table 1, while some species having very little proportion in the grassland or with no disease found were not listed. Eight of these listed species had significantly higher disease indexes in the fenced grassland compared with those in the overgrazed grassland (*P* < 0.05), with most of them being dominant or “common” species. However, there were two species of plants that had significantly lower disease indexes in the fenced grassland compared to the overgrazed grassland (*P* < 0.05) and there were two species showing no significant difference. Besides, two species were identified to be present only in overgrazed grassland, with indexes lower than 18.00. For plant species with a disease index higher than 18.00, eight species were found in the fenced grassland, while no species were found in the overgrazed grassland. Among these eight species, five are considered typical forage plants, including the dominant species, *Elymus nutans* which had a disease index around twice that observed in overgrazed grassland.

### Disease indexes of plants in the overgrazed grasslands without or with *A. inebrians* and fenced for one year (AO vs. AI)

As shown in Table 2, totally14 species were presented. Seven species having a significantly higher disease index in overgrazed grassland without *A. inebrians* than with *A. inebrians* present (*P* < 0.05). One species had no statistical significance in the two treatments whereas six species were diseased only in overgrazed grassland without *A. inebrians*. There were nine species with indexes higher than 20.00 in the overgrazed grassland without *A. inebrians*, but none in the overgrazed grassland with *A. inebrians*. Among these nine species, six were predominant forage grasses. The disease index of the dominant species, *E. nutans*, in overgrazed grassland without *A. inebrians* was approximately three times of that in the overgrazed grassland with *A. inebrians*.

### Disease indexes of plants in AO and in the overgrazed or fenced grasslands

**Figure 1:**
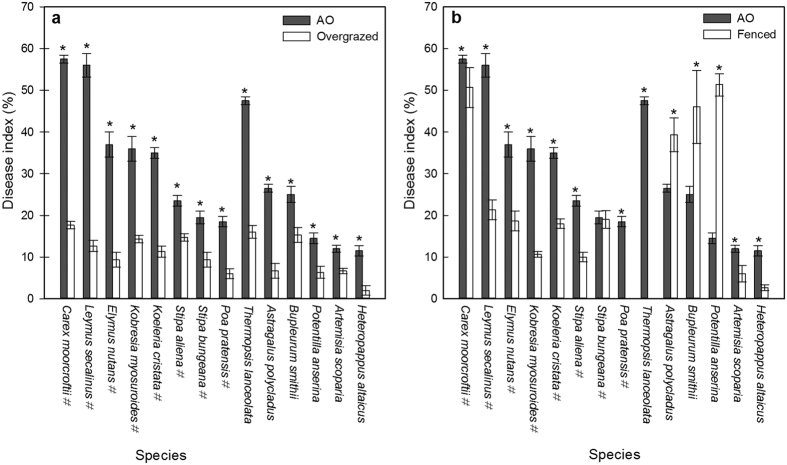
Comparison of disease status of plants in the overgrazed grassland without *A. inebrians* and fenced for one year (AO), and in the grasslands overgrazed or fenced for 18 years. (**a**) Comparison of disease indexes of plants in the overgrazed grassland without *A. inebrians* and fenced for one year, and in the grassland overgrazed for 18 years. (**b**) Comparison of disease indexes of plants in the grassland without *A. inebrians* and fenced for one year, and in the grassland fenced for 18 years. Notes: *indicates a significant difference at *P* = 0.05. ^#^indicates the plant species is a forage plant. Results are presented as means ± SE.

For comparing the disease indexes of plants in AO and in the overgrazed grassland, totally14 species were listed. All plants had higher disease indexes in AO than in the overgrazed grassland (*P* < 0.05) (Fig. 1a).

For comparison, the disease indexes of plants in AO and in the fenced grassland, totally14 species were presented. There were ten plant species having higher disease indexes in AO than in the fenced grassland (*P* < 0.05). Three plant species had higher indexes in the fenced grassland than in AO (*P* < 0.05). One species had no significant difference. Eight of the nine forage plant species had higher indexes in AO than in the fenced grassland (*P* < 0.05). In general, the disease indexes were higher in AO than in the fenced grassland (*P* < 0.05) (Fig. 1b).

### Disease indexes of plants in AI and in the overgrazed or fenced grasslands

**Figure 2:**
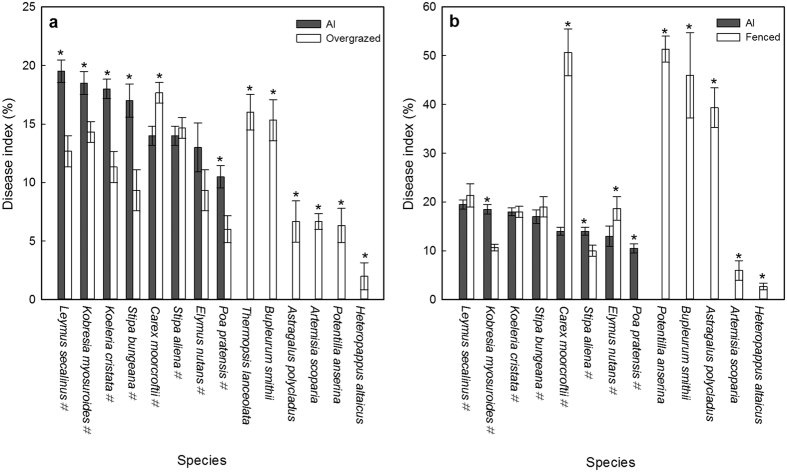
Comparison of disease status of plants in the overgrazed grassland with *A. inebrians* and fenced for one year (AI), and in the grasslands overgrazed or fenced for 18 years. (**a**) Comparison of disease indexes of plants in the overgrazed grassland with *A. inebrians* and fenced for one year, and in the grassland overgrazed for 18 years. (**b**) Comparison of disease indexes of plants in the overgrazed grassland with *A. inebrians* and fenced for one year, and in the grassland fenced for 18 years. Notes: *indicates a significant difference at *P* = 0.05. ^#^indicates the plant species is a forage plant. Results are presented as means ± SE.

For comparing the disease indexes of plants in AI and in the overgrazed grassland, totally14 species were displayed. Compared with the disease indexes of plants in the overgrazed grassland, five forage plant species had significantly higher indexes (*P* < 0.05); two forage plants had no significant difference; and one forage plant had a lower index (*P* < 0.05). Six other unpalatable plant species had lower indexes (*P* < 0.05) in AI (Fig. 2a).

For comparing the disease indexes of plants in AI and in the fenced grassland, totally13 species were presented. Compared with the fenced grassland, seven plant species had significantly lower disease indexes (*P* < 0.05); three species had no significant difference; and three had significantly higher disease indexes (*P* < 0.05) in AI (Fig. 2b).

### Disease indexes of *A. inebrians* in the six experimental sites

*Achnatherum inebrians* was seriously infected by fungal diseases in the six experimental sites, especially for powdery mildew and rust. The powdery mildew disease indexes of *A. inebrians* were higher than 50 in all the regions. The mean values were 51.60, 73.60, 62.40, 78.00, 64.00 and 97.20 in Yuzhong, Huining, Xiahe, Guinan, Haiyuan and Alxa, respectively. The mean indexes of rust were higher than 60, except for 46.80in Guinan. The mean values were 95.60, 82.00, 85.60, 46.80, 67.20 and 77.60 in Yuzhong, Huining, Xiahe, Guinan, Haiyuan and Alxa, respectively. The mean indexes of ergot were 40.00, 29.20 and 38.40 in Huining, Xiahe and Haiyuan, respectively. But no ergot was found in Yuzhong, Guinan and Alxa. The mean index of stem smut was 98.40 in Alxa, but was not found in the other five sites (Supplementary Information S2).

### Mycoparasitism rate of *Ampelomyces quisqualis* and *Sphaerellopsis filum*

The mycoparasitism rates of both *A. quisqualis* and *S. filum* on the diseased leaves of *A. inebrians* were found to be high in all the six sites, with the ranges of rates being 80.70–95.10% and 75.20–92.80% (Supplementary Information S3). The mean values of *A. quisqualis* were 94.50, 80.70, 90.50, 95.10, 80.70 and 87.70 in Yuzhong, Huining, Xiahe, Guinan, Haiyuan and Alxa, respectively. The mean values of *S. filum* were 81.40, 75.20, 77.80, 84.60, 92.80 and 89.10 in the six sites.

As identified, only one sub-family of arthropod, Aleocharinae (Staphylinidae), was fungivorous, and it was found only in the soil under tussock of *A. inebrians*, not in the overgrazed grassland without *A. inebrians*. The mean number of this insect was 2.12, 1.33 and 0.75 per block in Xiahe, Guinan and Alxa, respectively (not shown in Table).

## Discussion

There are serious overgrazing problems in many grasslands of China^1^,^2^,^3^. It has been reported that the level of disease incidence is relatively lower in grasslands heavily grazed^6^,^7^,^8^. Similarly, our results show that most plant species, including dominant and common species, had lower disease incidence in grassland overgrazed for 18 years compared to grassland that had been fenced for the same period (regarded as a healthy grassland in this study) (Table 1). This suggests that the levels of pathogenic fungi in overgrazed grassland is less than for fenced grassland. As such, mycophagous predators and mycoparasites of pathogens and other microorganisms maybe barely survive in overgrazed grassland leading to a subsequent lack of control for pathogenic species that can dramatically increase during pasture recovery (due to fencing).

Currently, fencing is a common way to help achieve grassland restoration^9^,^10^. As the overgrazed grassland is fenced or the grazing intensity is reduced for restoration, we can probably see more frequent plant disease occurrences in the future. Our study indicated that the disease indexes of forage plants were significantly higher in overgrazed grassland without *A. inebrians* and fenced for one year, compared with the grassland overgrazed for 18 years (*P* < 0.05) (Fig. 1a), and were also significantly higher than the disease indexes of forage plants in the grassland fenced for 18 years (*P* < 0.05) (Fig. 1b). On the contrary, when *A. inebrians* was present, the disease level of plants in the overgrazed grassland fenced for one year was significantly lower than the disease level of plants in the overgrazed grassland without *A. inebrians* and fenced for one year (*P* < 0.05) (Table 2). A possible explanation is that seriously diseased *A. inebrians* provided enough pathogens to support the mycophagous predators and mycoparasites when pathogen was lacking in the slightly diseased grassland. Much research has shown that mycophagous predators and mycoparasites can reduce the prevalence of pathogens^19^,^20^,^21^,^22^,^23^,^24^. Accordingly, these biocontrol organisms could inhibit the explosion of pathogens by consuming them when overgrazed grassland is fenced for restoration.

In the overgrazed grassland with *A. inebrians* and fenced for one year, the results indicated that the forage plants had higher disease indexes, but the unpalatable plants had lower disease indexes, compared with those in the grassland overgrazed for 18 years (Fig. 2a).When compared with the grassland fenced for 18 years, on the whole, the disease indexes were significantly lower in the overgrazed grassland with *A. inebrians* and fenced for one year, but the disease levels of forage plant species were very close in the two treatments (Fig. 2b).

These results indicate that disease levels are likely to increase in overgrazed grasslands after being fenced for restoration, but this outcome could be modified by diseased *A. inebrians*. Probably, it will gradually get to a similar level with the grassland fenced for 18 years treated as a healthy status. For a healthy grassland, there should be enough pathogens supporting mycophagous predators and mycoparasites. As a balance is achieved between them, the plant diseases can be kept at normal levels.

In contrast to the degraded grassland, *A. inebrians* can survive because of its toxicity to livestock that generally do not eat it^11^,^12^. Moreover, in contrast to a low disease incidence in overgrazed grassland, our results showed that *A. inebrians* were heavily infected by fungal diseases. These findings were consistent with the study by Li *et al*.^18^. We can see that the disease indexes of *A. inebrians* were significantly higher than other plants (as shown in Table 1). The resulted pathogens from *A. inebrians* provided a food source for their predators and parasites to live with. For example, the only mycophagous invertebrate, Aleocharinae, which consumes pathogens as food^25^ was found only in the soil block under the tussock of *A. inebrians*, but not in overgrazed grassland without *A. inebrians* in Xiahe, Guinan and Alxa (not shown in Table). In addition, our study showed that a lot of *A. quisqualis* and *S. filum* parasitising powdery mildew and rust, respectively, were found on the leaves of *A. inebrians*. *Ampelomyces quisqualis* has been found to be parasitic on more than 65 species of powdery mildew^26^,^27^ and used as biocontrol agent of powdery mildews (AQ10 Biofungicide; Ecogen Inc., Langhorne, PA, USA)^27^. *Sphaerellopsis filum* was reported to colonize 369 rust species^28^ and decreased the severity of rust by reducing spore production^28^,^29^,^30^,^31^. These fungivorous insect and mycoparasites are therefore beneficial in the control of plant diseases in overgrazed grasslands when they are fenced for restoration.

This study showed that the degenerated grassland without *A. inebrians* was susceptible to diseases when restored by fencing. Past research has suggested that increased biodiversity could decrease disease levels^32^,^33^,^34^, namely, a popular hypothesis is called “diversity-disease”. However, one review article indicated that even though biodiversity could generally reduce the prevalence of infectious diseases, the mechanism remained to be understood^35^. Our study indicates that the occurrence of plant diseases is probably determined by the balance in the pathogenic food chain. As a part of the food chain, pathogen, including fungi, bacteria and other microbes, not only can be a consumer but also can be consumed. When the food chain is broken, disease will have chances to breakout. Therefore, toxic and susceptible plants can be key species to control diseases in the grasslands.

## Methods

### Site description

We conducted this study at six different sites in northwestern China: Yuzhong county, Huining county and Xiahe county of Gansu province, Guinan county of Qinghai province, Haiyuan county of Ningxia provinceand Alxa Left Banner county of Inner Mongolia (Supporting Information S4). The geographical coordinates of these sites are presented in Supporting Information S5, and temperature and precipitation data in Supporting Information S6. The grassland type in Alxa Left Banner county of Inner Mongolia is very similar to that in Xinjiang, and both regions are widely distributed with *A. inebrians*. All of the experiments were conducted and samples collected in 2013. The *A. inebrians* samples in all locations were identified as 100% infected with fungal endophyte following the method of Bacon *et al*.^36^.

### Experimental design

Field investigations were conducted from August 22nd to September 21st in 2013, and samples were collected randomly in each site with methods mentioned below. Identification of plants, microorganisms and arthropods was carried out afterwards in the State Key Laboratory of Grassland Agro-ecosystems in Lanzhou University. Experimental plots were designed as presented in Supporting Information S7.

### Disease indexes of plants in grasslands fenced or overgrazed for 18 years (fenced vs. overgrazed grasslands)

This study compared the disease incidence in overgrazed or fenced grasslands in Xiahe county, i.e., Sangke grassland. The grazing rate in the overgrazed grassland was approximately 10 sheep per hectare. No livestock were grazed in the fenced grassland. The grasslands had been either overgrazed with livestock or fenced to exclude grazing for at least 18 years. We chose four blocks (10 m × 10 m) for each treatment. Using the five-point sampling method (the crossing point of two cross wires connecting 4 corners of a block and the 4 intermediate points between the center and each of the 4 corners), we collected plant samples and randomly removed 10 leaves or stems per plant species to record the disease rate and severity. The severity classification for each disease is presented in Supporting Information S8.

The formula for calculating the disease severity is specified as equations (1):


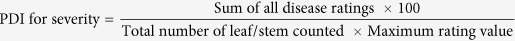


where PDI meansthe percentage of disease index.

In order to calculate the importance of different plant species, four blocks (1 m × 1 m) were chosen for each treatment, the biomass of each species was oven-dried at 75 °C for 48 hours to a constant weight. Then the importance of species was expressed by the percentage of dry weight.

### Disease indexes of plants in overgrazed grassland without or with *A. inebrians* and fenced for one year (AO vs. AI)

The same method mentioned above was applied to investigate plants disease status in AO and AI. These two treatments were fenced from January to late October of 2013 to examine the effects of seriously diseased *A. inebrians* on the disease status of grassland after fencing. Then the disease indexes of plants in AO and AI were compared with that in the grasslands fenced or overgrazed for 18 years, respectively.

### Diseases on *A. inebrians* and mycoparasites on diseased leaves

To investigate the diseases of *A. inebrians*, all thesix sites were selected. In each site, the five-point sampling method was employed, and 10 leaves from each plant were used to record the disease rate and severity on *A. inebrians*. The presence of two mycoparasites on diseased leaves of *A. inebrians* was investigated in this study. One parasitized rust sori and the other was parasitic on powdery mildew.The mycoparasitism rate on rust sori was the percentage of rust sori that was colonised. The mycoparasitism rate on powdery mildew was the infection percentage, i.e., the area infected by the mycoparasite divided by the area infected by powdery mildew on the leaf.

### Effects of *A. inebrians* on fungivorous arthropods

Three sites were selected for this study, specifically, the grassland dominated by *Elymus nutans* in Xiahe, the wetland-type grassland dominated by *Kobresia capillifolia* in Guinan, and the arid grassland dominated by *Pennisetum centrasiaticum* in Alxa Left Banner. *A. inebrians* plants were found at these three sites. For each site, 30 soil blocks (0.4 m in diameter ×0.2 m in depth) were dug out, including 15 soil blocks for the overgrazed grassland without *A. inebrians*, and 15 under tussock of *A. inebrians*. These blocks were crushed to collect arthropods, which were brought back to the laboratory to identify whether they were fungivorous arthropods.

### Data analysis

Data were analyzed using SPSS 17.0 for Windows. The T-test for independent-samples was used to compare: 1) disease status of plants in the overgrazed or fenced grasslands; 2) disease status of plants in the overgrazed grasslands without or with *A. inebrians* after being fenced for one year (AO vs. AI); 3) disease status of plants in AO with that in the overgrazed or fenced grasslands; and 4) disease status of plants in AI with that in the overgrazed or fenced grasslands. Disease index of *A. inebrians* and mycoparasite rates were analyzed using one way ANOVA, and the number of mycophagous arthropods and dry weight of plant species were presented with means and standard errors (SE). The statistical significance was defined at the 95% confidence level (alpha = 0.05). All mean values were presented with ±1 SE.

## Additional Information

**How to cite this article**: Yao, X. *et al*. Modification of Susceptible and Toxic Herbs on Grassland Disease. *Sci. Rep.* **6**, 30635; doi: 10.1038/srep30635 (2016).

## Supplementary Material

Supplementary Information

## Figures and Tables

**Table 1:** Indexes of plant diseases in grasslands fenced vs. overgrazed for 18 years.

Species	Disease	Fenced	Overgrazed	*t*	*P*
Forage plants					
*Carex moorcroftii*	Rust	50.67 ± 4.81	17.67 ± 0.88	6.75	0.003
*Leymus secalinus*	Leaf spot	21.33 ± 2.40	12.67 ± 1.33	3.15	0.034
*Elymus nutans*	Leaf spot	18.67 ± 2.40	9.33 ± 1.76	3.13	0.035
*Stipa bungeana*	Leaf spot	19.00 ± 2.08	9.67 ± 1.45	3.68	0.026
*Koeleria cristata*	Leaf spot	18.00 ± 1.15	11.33 ± 1.33	3.78	0.019
*Kobresia myosuroides*	Rust	10.67 ± 0.67	14.33 ± 0.88	−3.31	0.029
*Stipa aliena*	Leaf spot	10.00 ± 1.15	14.67 ± 0.88	−3.21	0.033
*Poa pratensis*	Leaf spot	/	6.00 ± 1.15		
Unpalatable plants					
*Potentilla anserina*	Rust	51.33 ± 2.67	6.33 ± 1.45	14.81	0.000
*Bupleurum smithii*	Rust	46.00 ± 8.72	15.33 ± 1.76	3.44	0.026
*Astragalus polycladus*	Leaf spot	39.33 ± 4.06	6.67 ± 1.76	7.38	0.002
*Artemisia scoparia*	Leaf spot	6.00 ± 2.00	6.67 ± 0.67	−0.31	0.768
*Heteropappus altaicus*	Leaf spot	2.67 ± 0.67	2.00 ± 1.15	0.50	0.643
*Thermopsis lanceolata*	Leaf spot	/	16.00 ± 1.53		

Note: “/” indicates the plant species was not found in the treatment; “Fenced” means the grassland was fenced for 18 years; “Overgrazed” means the grassland was overgrazed for 18 years; “*P* < 0.05” means the difference is significant.

**Table 2:** Disease indexes of plants in the overgrazed grassland without or with *A. inebrians* and fenced for one year (AO vs. AI).

Species	Disease	AO	AI	*t*	*P*
Forage plants					
*Carex moorcroftii*	Rust	57.50 ± 0.96	14.00 ± 0.82	34.57	0.00
*Leymus secalinus*	Rust	56.00 ± 2.83	19.50 ± 0.96	12.22	0.00
*Elymus nutans*	Leaf spot	37.00 ± 3.00	13.00 ± 2.08	6.57	0.001
*Kobresia myosuroides*	Leaf spot	36.00 ± 2.94	18.50 ± 0.96	5.65	0.006
*Koeleria cristata*	Leaf spot	35.00 ± 1.29	18.00 ± 0.82	11.13	0.00
*Stipa aliena*	Leaf spot	23.50 ± 1.26	14.00 ± 0.82	6.33	0.001
*Stipa bungeana*	Leaf spot	19.50 ± 1.50	16.00 ± 1.41	1.70	0.14
*Poapratensis*	Leaf spot	18.50 ± 1.26	10.50 ± 0.96	5.06	0.002
Unpalatable plants					
*Thermopsis lanceolata*	Leaf spot	47.50 ± 0.96	0.00 ± 0.00		
*Astragalus polycladus*	Leaf spot	26.50 ± 0.96	0.00 ± 0.00		
*Bupleurum smithii*	Leaf spot	25.00 ± 1.91	0.00 ± 0.00		
*Potentilla anserina*	Leaf spot	14.50 ± 1.26	0.00 ± 0.00		
*Artemisia scoparia*	Leaf spot	12.00 ± 0.82	0.00 ± 0.00		
*Heteropappus altaicus*	Leaf spot	11.50 ± 1.26	0.00 ± 0.00		

Note: 0.00 ± 0.00 indicates no disease was found; “AO” means the overgrazed grassland without *A. inebrians* and fenced for one year; “AI” means the overgrazed grassland with *A. inebrians* and fenced for one year; “*P* < 0.05” means the difference is significant.
