# Supplementary material for: Modification of Susceptible and Toxic Herbs on Grassland Disease
Source: Sci Rep. 2016 Sep 16;6:30635. doi: 10.1038/srep30635 (PMC5025904; doi:10.1038/srep30635)
Supplement: Supplementary Information [file srep30635-s1.pdf]

Supplementary Information

Title: Modification of Susceptible and Toxic Herbs on Grassland Disease

Author list: Xiang Yao[1], Yubing Fan[2], Qing Chai[1], Richard D. Johnson[3], Zhibiao Nan[1], Chunjie Li[1,*]

S 1 Percentage of dry weight of main plant species (%) in overgrazed and fenced grasslands.

Species	Overgrazed	Species	Fenced
Forage plants			
Stipa bungeana	24.68±1.22	*Elymus nutans*	36.08±1.36
Kobresia myosuroides	8.69±0.45	*Leymus secalinus*	18.45±2.49
Leymus secalinus	4.64±1.00	*Koeleria cristata*	14.65±2.84
Koeleria cristata	2.97±0.31	*Carex moorcroftii*	3.53±0.42
Poa pratensis	2.73±0.62	*Stipa bungeana*	1.51±0.70
Elymus nutans	2.45±0.67	*Kobresia myosuroides*	1.30±0.26
Carex moorcroftii	2.12±1.07	*Stipa aliena*	0.73±0.10
Stipa aliena	1.40±0.58		
Upalatable plants			
Artemisia scoparia	10.74±1.91	*Bupleurum smithii*	5.07±0.19
Heteropappus altaicus	6.43±0.91	*Artemisia scoparia*	3.64±0.43
Thermopsis lanceolata	5.00±0.26	*Heteropappus altaicus*	1.84±0.07
Bupleurum smithii	4.85±0.18	*Potentilla anserina*	0.98±0.12
Astragalus polycladus	3.43±0.39	*Astragalus polycladus*	0.73±0.60
Potentilla anserina	0.87±0.28		

S 2 Disease indexes of *A. inebrians* in the six experimental sites

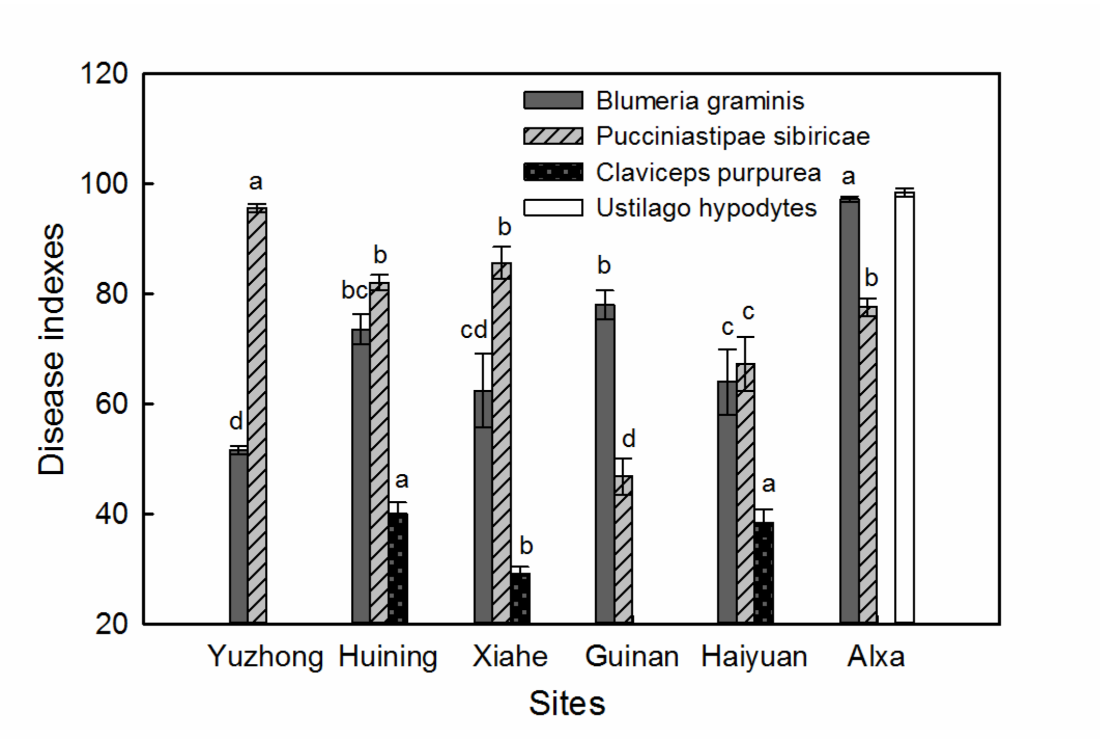

Note: *Blumeria graminis* was identified on leaves; *Pucciniastipae-sibiricae* was on leaves; *Claviceps purpurea* was on inflorescences; and*Ustilago hypodytes* was on stems. Index of the same disease was compared in six sites and the significance was labelled. Columns with the same color sharing the same letters are not significantly different from each other, significant difference at P = 0.05.

S 3 Mycoparasitism rate of *Ampelomyces quisqualis* and *Sphaerellopsis filum*

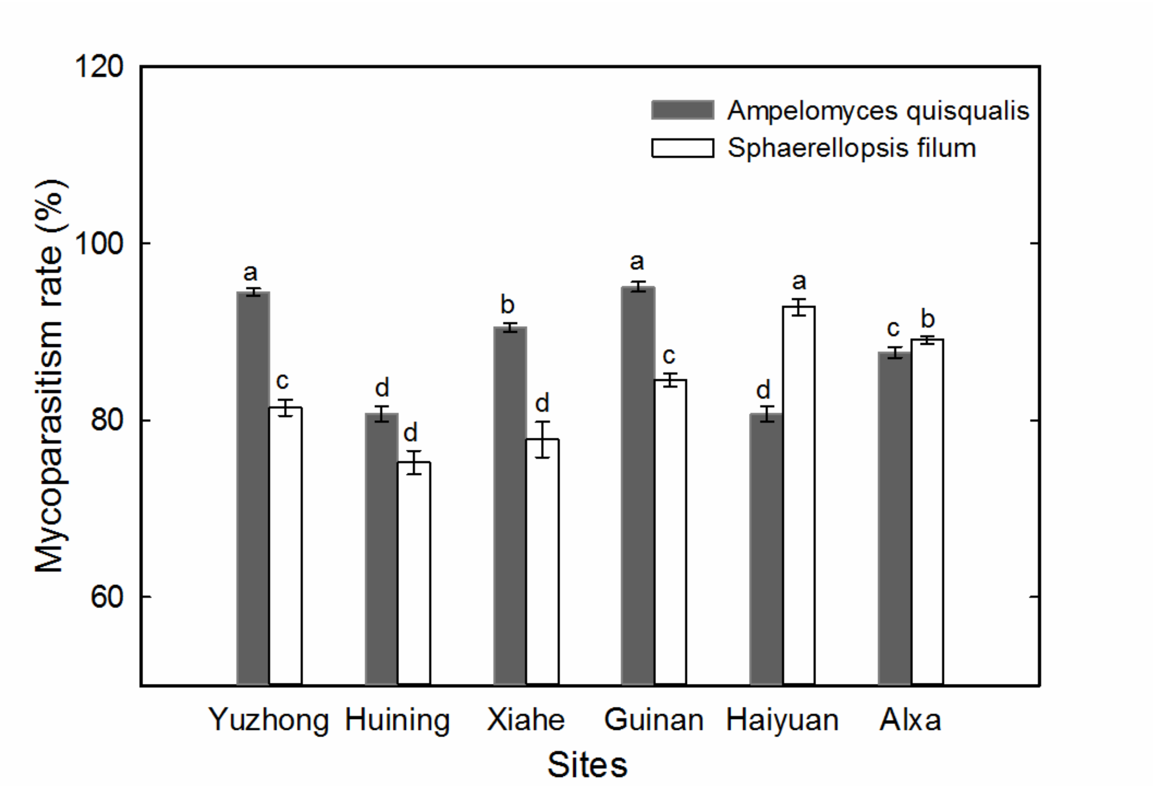

Note: mycoparasitism rate of the same mycoparasite was compared in six sites and the significance was labelled. Columns with the same color sharing the same letters are not significantly different from each other, significant difference at P = 0.05.

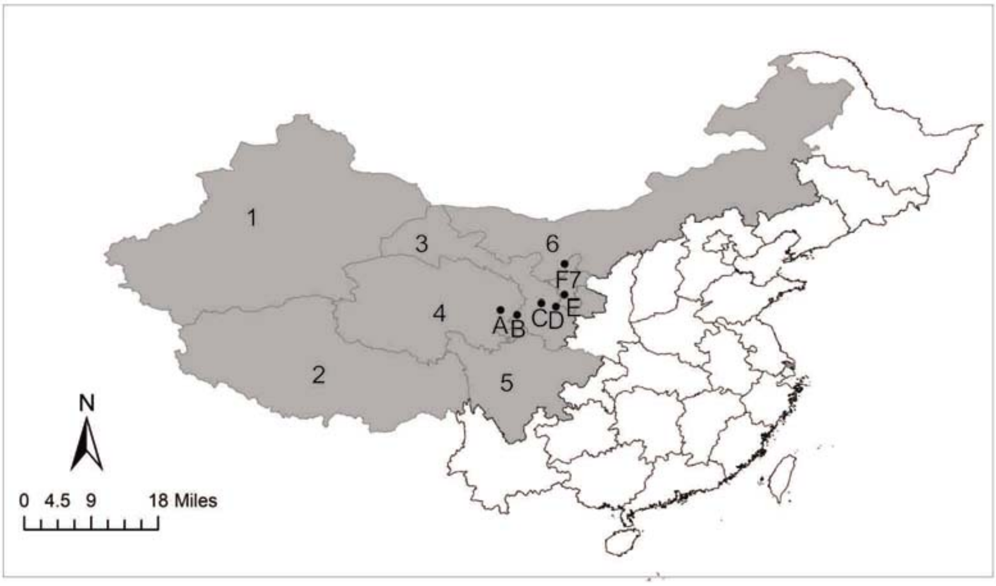

S 4 Distribution of *Achnatherum inebrians* and the locations of study area. Gray color indicates provinces distributed with *Achnatherum inebrians*. 1: Xinjiang Uygur autonomous region; 2: Tibet autonomous region; 3: Gansu province; 4: Qinghai province; 5: Sichuan province; 6: Inner Mongolia autonomous region; 7: Ningxia autonomous region. Black points indicate six experimental sites. A: Guinan county, Qinghai province; B: Xiahe county, Gansu province; C: Yuzhong county, Gansu province; D: Huining county, Gansu province; E: Haiyuan county, Ningxia autonomous region; F: Alxa left banner, Inner Mongolia autonomous region. This map was drew with ArcGIS, ArcMap 10.2 (http://www.arcgis.com/features/).

S 5 Geographical coordinates of experimental sites

Sites	Latitude	Longitude	Elevation (m)
Yuzhong, Gansu	35°56'	104°08'	1774
Huining, Gansu	35°42'	105° 09'	1845
Xiahe, Gansu	35°07'	102° 26'	3077
Guinan, Qinghai	35° 28'	101° 16'	3572
Haiyuan, Ningxia	36°30'	105° 45'	1809
Alxa, Inner Mongolia	38° 39'	105° 46'	1987

S 6 Meteorological data

Mean temperature (℃) of sample areas in recent 5 years

Year	Yuzhong	Huining	Xiahe	Guinan	Haiyuan	Alxa
2008	7.02	9.50	2.90	2.49	7.89	8.48
2009	7.69	10.06	3.62	3.18	8.44	9.59
2010	7.45	9.82	3.83	3.35	8.21	9.04
2011	7.15	9.67	3.17	2.64	7.26	8.62
2012	6.64	9.48	3.02	2.29	7.19	8.51

Mean precipitation (mm) of sample areas in recent 5 years

Year	Yuzhong	Huining	Xiahe	Guinan	Haiyuan	Alxa
2008	386.9	202.1	552.3	456.4	283.5	291.2
2009	299.8	159.2	413.1	506.1	277.7	134.8
2010	332.3	153.1	523.5	402.3	353.5	159.4
2011	262.8	200.2	582.7	455.2	345.7	171.0
2012	395.2	221.9	646.1	591.2	431.1	233.4

S 7 Design of experimental plots

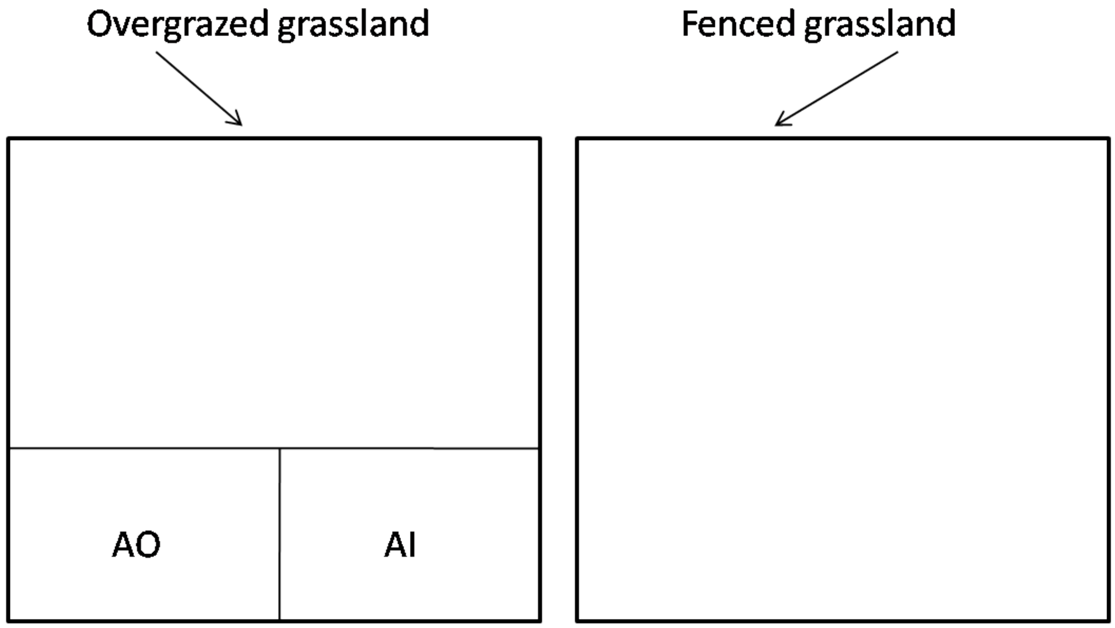

Note: "Overgrazed grassland" means grassland overgrazed for 18 years; "Fenced grassland" means grassland fenced for 18 years; "AO" means overgrazed grassland without *Achnatherum inebrians* fenced for one year; "AI" means overgrazed grassland with *A. inebrians* fenced for one year.

S 8 Severity classification of all kinds of diseases

Severity classification of rust	
0	No disease
1	Percentage of leaf covered by sorus< 1%
2	Percentage of leaf covered by sorus 1%-5%
3	Percentage of leaf covered by sorus 5%-25%
4	Percentage of leaf covered by sorus 25%-50%
5	Percentage of leaf covered by sorus> 50%
Severity classification of powdery mildew	
0	No disease
1	Percentage of leaf covered by mycelium < 5%
2	Percentage of leaf covered by mycelium 5%-25%
3	Percentage of leaf covered by mycelium 25%-50%
4	Percentage of leaf covered by mycelium 50%-75%
5	Percentage of leaf covered by mycelium > 75%
Severity classification of stem smut	
0	No disease
1	Percentage of stem covered by sorus< 5%
2	Percentage of stem covered by sorus 5%-30%
3	Percentage of stem covered by sorus 30%-60%
4	Percentage of stem covered by sorus 60%-90%
5	Percentage of stem covered by sorus> 90%
Severity classification of ergot	
0	No ergot
1	Amount of ergot ≤ 2
2	2 < amount of ergot ≤ 5
3	5 < amount of ergot ≤ 8
4	8 < amount of ergot ≤ 11
5	Amount of ergot > 8
Severity classification of leaf spot disease	
0	No disease
1	Percentage of leaf covered by leaf spots < 1%
2	Percentage of leaf covered by leaf spots 1%-5%
3	Percentage of leaf covered by leaf spots 5%-25%
4	Percentage of leaf covered by leaf spots 25%-50%
5	Percentage of leaf covered by leaf spots > 50%
